# Endoscopic Transnasal Approach for Cholesterol Granuloma of the Petrous Apex

**DOI:** 10.1155/2015/481231

**Published:** 2015-07-21

**Authors:** Mohammad Samadian, Nader Akbari Dilmaghani, Navid Ahmady Roozbahany, Navid Farzin, Mohammad Bahadoram

**Affiliations:** ^1^Department of Neurosurgery, Loghman Hakim Hospital, Shahid Beheshti University of Medical Sciences, Tehran, Iran; ^2^Department of Otolaryngology, Loghman Hakim Hospital, Shahid Beheshti Medical University, Tehran, Iran; ^3^Medical Student Research Committee and Social Determinant of Health Research Center, Ahvaz Jundishapur University of Medical Sciences, Ahvaz, Iran

## Abstract

Cholesterol granulomas are rare round or ovoid cysts. They contain cholesterol crystals surrounded by foreign bodies of giant cells and are characterized by chronic inflammation. Large cholesterol granuloma can compress surrounding tissue especially cranial nerves. There are several types of surgery for the resection of cholesterol granuloma. We describe 4 cases of cholesterol granuloma operated on via transnasal endoscopic approach. In this report, we describe radiologic and pathologic features of this lesion and explain the advantages and disadvantages of transsphenoidal endoscopic approach for these rare lesions.

## 1. Introduction

Cholesterol granulomas (CG) (mucocele, cholesterol cyst) are rare and expansile, round, or ovoid cysts containing cholesterol crystals surrounded by foreign body giant cells and chronic inflammation, all contained within a thick fibrous capsule [1]. The lesion is secondary to chronic obstruction of air cells within the petrous pyramid [2]. Cholesterol granuloma was first described in 1894 [3], It is commonly encountered in the middle ear and mastoid air cells. It occurs after the obstruction of the normally aerated spaces due to associated diseases such as otitis media [3].

Most lesions are clinically silent until they adhere to cranial nerves. Further, this lesion can present with hearing loss, imbalance, facial weakness, and/or diplopia [3]. Principal treatment is surgical drainage and permanent aeration to prevent recurrence. One accepted surgical treatment of CGs is by way of ventilation tubes through a subcochlear or infralabyrinthine route when possible. This approach has the advantage of hearing conservation. Occasionally, with far medial lesions, a transsphenoidal route of drainage is elected [1, 3]. Although drainage procedures are often effective for a while, the ongoing secretion of bloody sludge often clogs the drainage tract. Recurrence on long-term follow-up ranges from 12 to 60% of cases [1, 3]. According to a study by Oyama et al. published in 2007, most of the cases were treated via the transtemporal or middle fossa approach [4]. We describe 4 cases that were operated via Endoscopic Transrostral-Transsphenoidal Approach.

## 2. Case Report

**Case 1 .**

A 28-year-old woman presented with a 6-month history of intermittent left hemicranial headache and diplopia. The patient had no history of head trauma or otologic problem but she had type 1 familial hyperlipidemia. Past surgical history was negative. Physical examination demonstrated right-sided sixth nerve palsy and mild left side hearing loss. The remainder of the exam was unremarkable.

**Figure 1:**
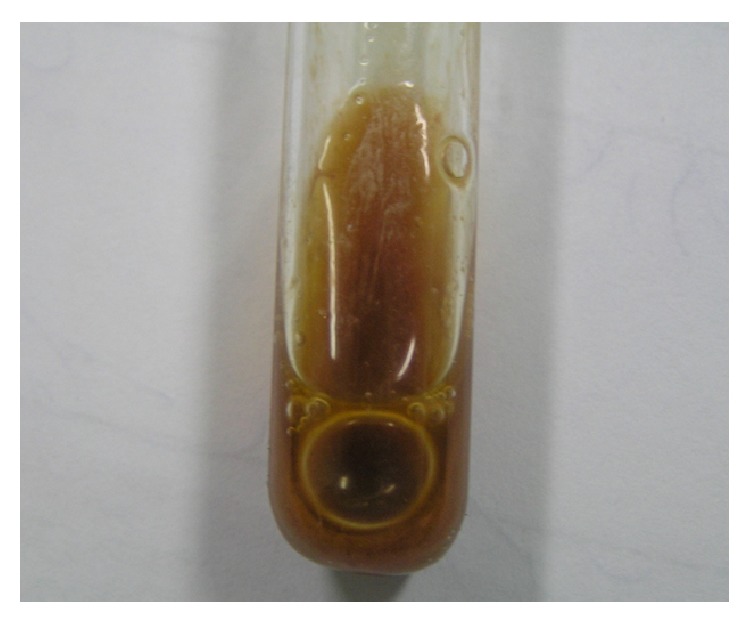
Golden-brown fluid drained from the cyst during surgery.

**Figure 2:**
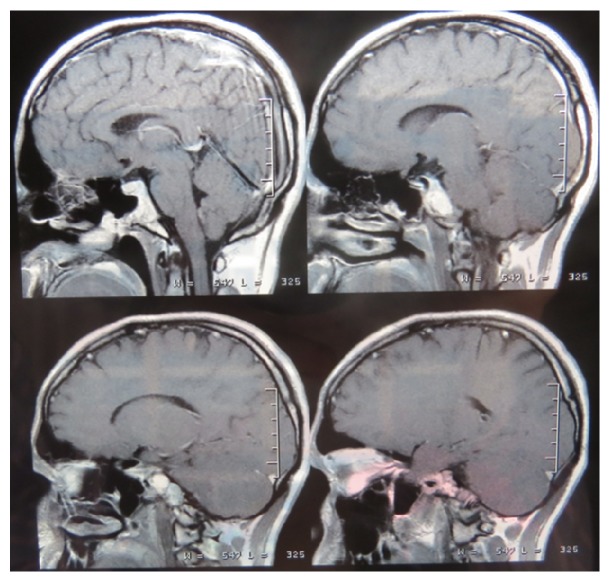
T1 weighted magnetic resonance imaging scans show resolution of the lesion.

Magnetic resonance imaging (MRI) revealed a large left petrous apex mass abutting the sphenoid sinus. The mass was hyperintense on both T1 and T2 weighted images. A CT scan of petrous bone and skull base showed an expansile mass of the left petrous apex with bone remodeling of the clivus and skull base, without contrast-induced enhancement. She was referred to our center for treatment of her skull base lesion. The lesion was separated from the posterior sphenoid sinuses by a thin layer of bone. Wide access to the cyst cavity was not possible without disruption of vital structures. Endoscopic drainage and resection of the cyst wall of the cholesterol granuloma were performed through the left nostril. With the assistance of the endoscope, the sphenoid septum was grabbed after the removal of the sphenoid mucosa. The sphenoid mucosa being placed on the rostrum at the level of the sphenoid sinus ostium, we managed to remove it without scarifying the sphenoid sinus ostium. With straight and angled endoscopes, golden-brown fluid and debris were removed, and the cyst was opened draining a brown liquid (Figure 1) and widely marsupialized. Exposed dura remained intact. A silicone drainage tube was placed in the opening window for three weeks. Total operative time was under 1 hour, and the patient tolerated the procedure well. This case was reported previously in Turkish Neurosurgery journal in 2009 by us [2]. Post-op image is shown (Figure 2).

**Case 2 .**

A 43-year-old male presented with a 3-month history of diplopia and nonpulsatile and positional headache. He experienced mild paresthesia in the right side of the face. He had no history of trauma or otologic intervention. His past medical history was negative. Right side sixth nerve palsy was detected in neurologic examination but other examinations for cranial nerves or other systems were unremarkable.

**Figure 3:**
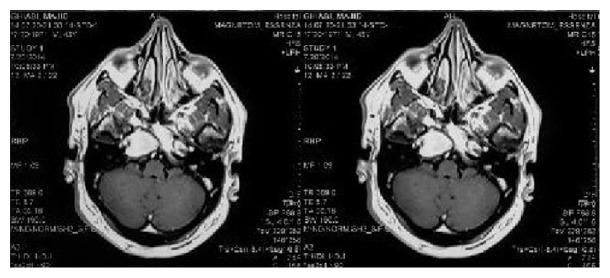
Axial post-GAD MRI showing the cholesterol granuloma involving the left petrous apex, adjacent to carotid artery.

**Figure 4:**
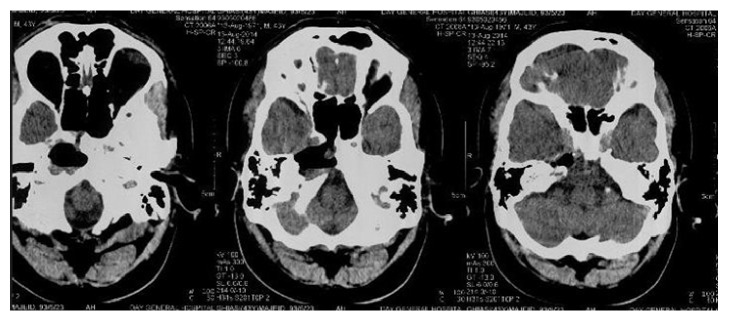
Axial CT scan shows acceptable aeration of right sphenoid sinus and previous location of the cyst.

CT scan revealed a hypodensity in the medial of right temporal lobe and with invasion of petrous apex and right upper clivus. There was a hyperintense mass lesion in both T1 and T2 sequences of MRI (Figure 3). Brain CT angiography was negative for vascular anomaly. The patient underwent endoscopic transnasal procedure like the previous case with the cooperation of an otolaryngologist but through the right nostril. After the removal of sphenoid sinus mucosa, bony layer of sinus was resected and cyst was opened and golden-brown liquid aspirated. After irrigation, the intact dura was exposed. In this case, we did not use silicon tube. Histopathologic study on the obtained fluid sample was compatible with cholesterol granuloma. The patient was discharged 4 days after surgery. Three months' follow-up brain CT scan revealed acceptable aeration of right sphenoid sinus and pervious location of cyst (Figure 4).

**Case 3 .**

**Figure 5:**
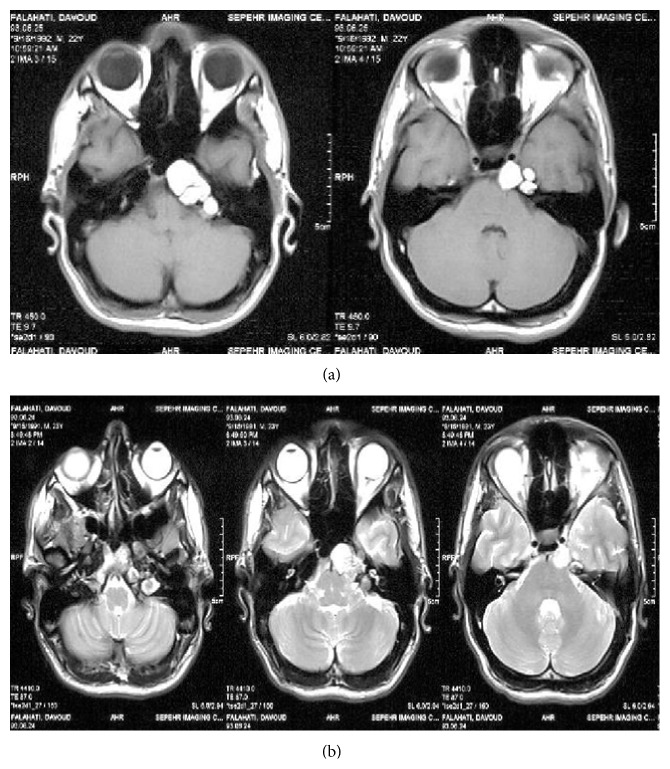
(a) T1 weighted MRI shows the cholesterol granuloma involving the right petrous apex. (b) Axial T2 weighted MRI shows the residual tumor at petrous apex adjacent to right carotid artery.

A 22-year-old male referred to our clinic with a complaint of diplopia at looking to left side from 2 weeks earlier. He had no history of trauma, headache, or visual disturbance. His past medical history was negative for medical and surgical problems. Sixth nerve palsy was detected in neurological examination. Other cranial nerves were intact and systemic examinations were unremarkable. MRI demonstrated a multicystic hyperintense lesion in apex of petrous bone with mass effect on midbrain (Figure 5(a)). The bone window CT scan images showed expansile mass on the left petrous apex with bone remodeling of the skull base. The same as previous cases, the endoscopic transnasal approach was selected for the patient. After resection of sphenoid sinus mucosa in lateral aspect of sphenoid sinus, a cystic lesion appeared. The cyst was dissected and golden-brown fluid was drained and sample was obtained. The pathology report was cholesterol granuloma. He was discharged 3 days after surgery but he still had a complaint of diplopia. On follow-up, 1 month after surgery, diplopia had regressed and the left sixth nerve examination was normal but MRI demonstrated a small cystic lesion in site of surgery that recommended following up (Figure 5(b)).

**Case 4 .**

A 28-year-old woman was referred to our clinic by an ophthalmologist because of a 6-month history of right eye visual loss and headache. MRI showed a large cystic lesion in medial temporal lobe on the right side. It extended from petrous apex to sphenoid sinus that was hyperintense in T1 and isointense on T2. Via transsphenoidal endoscopic approach, after sphenoidectomy, oily material was obtained. After drainage of gray fluid, the wall of cavity was gently excised from dura. Histology confirmed a cholesterol granuloma of the petrous apex. There was no evidence of recurrence or reaccumulation on 6 months' follow-up.

## 3. Discussion

Petrous apex cholesterol granulomas results from obstruction of the normal aeration of petrous air cells. Another location where cholesterol granuloma has been detected is pneumatic pathway of temporal bone [5]. The lesion is usually silent until tumor size becomes large enough to compress cranial nerves. Huge cholesterol granuloma can present with impaired hearing, balance, speech, and swallowing [3]. Contralateral involvement of cranial nerves may be detected and this can cause false localization of the tumor. Cholesteatoma is a differential diagnoses of cholesterol granuloma in this region. Cholesterol granuloma is often seen as a round mass with defined border in CT scan which can be isodense, slightly hyperdense, or mixed. Cholesteatoma is displayed as a soft hemogenic tissue with bone degeneration in CT scan [6]. In MRI, Cholesterol granuloma generates a high signal in both T1 and T2 imaging which is enhanced after introducing contrast. However, the granulation and inflammatory tissue of mucosal cholesteatoma delivers iso-to-hyposignal in T1 imaging and gives hypersignal in T2 and cannot be enhanced even by introducing high contrast [7]. Reports suggest a good DW image in MRI for diagnosing cholesteatoma which is seen as high signal [8].

Principle treatment in symptomatic cholesterol granuloma is surgical debridement and establishing a permanent pathway to aeration to avoid recurrence [4]. Middle fossa approach and transcochlear, translabyrinthine, and infralabyrinthine and subcochlear approach have been used for surgical treatment of CG [9]. Eisenberg et al. reported recurrence rate of 60% for the above approaches [10].

Petrous apex (PA) is divided into three regions: superior, anterior-inferior, and posterior-inferior. In contemporary approaches for cholesterol granuloma of PA, in case of performing transnasal approach as extended we can reach not only the superior region but also the anterior-inferior and posterior-inferior regions.

In transcanal infracochlear approach, we do not always have access to the superior part but this approach is suitable for accessing PA anterior-inferior and partly posterior-inferior regions. Regarding radiologic studies, endoscopic transnasal approach opens a wider window than transcanal infracochlear approach [11]. Furthermore, translabyrinthine approach could be appropriate in cases where nerve VIII function is lost and tumor has advanced to the posterior-inferior area which is located in temporal region. Overall, considering the patient's hearing condition and the involved neurovascular factors in this area, an approach should be picked among endonasal, translabyrinthine, and medial fossa. In cases with complete hearing loss, translabyrinthine approach is acceptable and in cases without hearing impairment transcanal infracochlear approach is a good approach [12, 13]. In our reported patients there was no sensorineural impairment in cranial nerve VIII in any of the patients and this finding was corroborated in ABR as well. This is the reason why we chose transnasal approach for these patients.

Montgomery used transsphenoidal approach for the first time in 1977 for the drainage of cholesterol granuloma via an incision near the medial cantus [14]. In 1994, Fucci et al. applied nasal endoscope and gained access to petrous apex via transnasal-transsphenoidal approach [15]. Georgalas and colleagues presented 5 cases of cholesterol granuloma invading petrous apex via transnasal-transsphenoidal approach. They expressed that endoscopic transsphenoidal approach had advantages such as shorter duration, being less technically challenging, less morbidity due to shortened hospitalization time, and considerable decrease in the rate of facial weakness and hearing loss. Transient epistaxis is a rare complication [3]. The risk of optic nerve injury is low since the petrous apex location is at floor level of the sphenoid sinus and far from optic nerve [16]. Endoscopic visualization allows thorough removal of debris that consist of hemosiderin sediment and concretions that reduce the osmotic effect, leading to the accumulation of more debris [17]. Patients treated by ETSS can easily be followed up on an out-patient basis with a fiberscope, and where necessary, drainage of the cyst can be reestablished [3]. We used transrostral-transsphenoidal surgery in our patients to lessen the possibility of damage to sphenoid sinus ostium while preserving the sphenoid sinus function.

## 4. Conclusion

Regarding the advances in instruments in neuroendoscopy and characteristics of cholesterol granuloma including extradural and cystic lesions, we found transsphenoidal approach to be the best approach for the drainage of cholesterol granuloma. Endoscopic approach has lower risks for hearing function defect and other possible intracranial complications. This method provides wide access to the cyst, outpatient follow-up in office, and, if necessary, reopening with little morbidity.
